# Prenatal lead exposure is associated with decreased cord blood DNA methylation of the glycoprotein VI gene involved in platelet activation and thrombus formation

**DOI:** 10.1093/eep/dvv007

**Published:** 2015-11-27

**Authors:** Karin Engström, Filip Rydbeck, Maria Kippler, Tomasz K. Wojdacz, Shams Arifeen, Marie Vahter, Karin Broberg

**Affiliations:** ^1^ Division of Occupational and Environmental Medicine, Lund University, Lund, Sweden; ^2^ Unit of Metals and Health, Institute of Environmental Medicine, Karolinska Institutet, Stockholm, Sweden; ^3^ International Centre for Diarrhoeal Disease Research Bangladesh (ICDDR,B), Dhaka, Bangladesh

**Keywords:** cardiovascular disease, DNA methylation, epigenetic regulation, GPVI, Pb, thrombosis, differently methylated region

## Abstract

Early-life lead exposure impairs neurodevelopment and later exposure affects the cardiovascular system. Lead has been associated with reduced global 5-methylcytosine DNA methylation, suggesting that lead toxicity acts through epigenetic mechanisms. The objective of this study is to clarify how early-life lead exposure alters DNA methylation of specific genes, using an epigenomic approach. We measured lead concentrations in urine [gestational week (GW), 8] and erythrocytes (GW 14), using inductively coupled plasma mass spectrometry, for 127 pregnant mothers recruited in the MINIMat food and supplementation cohort in rural Bangladesh. Cord blood DNA methylation was analyzed with the Infinium HumanMethylation450K BeadChip, and top sites were validated by methylation-sensitive high-resolution melt curve analysis. Maternal urinary lead concentrations (divided into quartiles) showed significant (after adjustment for false discovery rate) inverse associations with methylation at nine CpGs. Three of these sites were in the 5′-end, including the promoter, of glycoprotein IV (*GP6*); cg18355337 (*q* = 0.029, *β* = −0.30), cg25818583 (*q* = 0.041, *β* = −0.18), and cg23796967 (*q* = 0.047, *β* = −0.17). The methylation in another CpG site in *GP6* was close to significant (cg05374025, *q* = 0.057, *β* = − 0.23). The erythrocyte lead concentrations (divided into quartiles) were also inversely associated with CpG methylation in *GP6*, although this was not statistically significant after false discovery rate adjustments. Eight CpG sites in *GP6* constituted a differentially methylated region in relation to urinary lead (*P* = 0.005, *q* = 0.48) and erythrocyte lead (*P* = 0.007, *q* = 0.46). In conclusion, we found that moderate prenatal lead exposure appears to epigenetically affect GP6, a key component of platelet aggregation and thrombus formation, suggesting a novel link between early lead exposure and cardiovascular disease later in life.

## Introduction

Lead, one of the environmental chemicals most toxic to developing children, readily passes the placenta and reaches the fetus [1], and primarily impairs neurodevelopment of the offspring [2–5]. In fact, recent risk assessments showed that lead toxicity appears to have no exposure threshold [4, 6–8]. Although oxidative stress and interaction with essential elements like calcium and zinc are often considered to be the primary mechanisms of lead toxicity [9, 10], recent research has suggested other mechanisms. Interference with regulation of gene expression through alteration of 5-cytosine DNA methylation may have particular importance for lead’s long-lasting effects [11, 12]. The few studies that address epigenetic effects of lead have mainly focused on methylation in retrotransposons (Alu and LINE1 families), which compose a large part of the human genome (∼25%) and function as a proxy for global methylation. Wright et al. [13] reported an inverse association between methylation of LINE1, but not Alu, and lead concentrations in patella, but not in tibia or blood (4.1 ± 2.4 µg/dl) in 517 elderly men from the USA. Also, post-partum lead concentrations in the patella of selected Mexican women were inversely associated with DNA methylation of LINE1 in cord blood of 103 newborns, and lead in tibia was inversely associated with methylation of Alu [14]. No associations with cord blood lead (6.6 ± 2.7 µg/dl) were observed. Li et al. [15] found that the methylation of LINE1 was lower in exposed workers from a battery plant (blood lead concentrations 21.2 ± 6.3 µg/dl) compared with controls (3.7 ± 1.8 µg/dl). They also exposed kidney cells to lead and found that methylation of LINE1 was inversely associated with lead exposure. Thus, lead appears to modify repetitive DNA sequences by hypomethylation. The aim of this study was to clarify how lead exposure early in life alters DNA methylation of specific genes, based on an epigenome-wide approach.

## Results

Descriptive data are listed in Table 1. The median body mass index was 20 kg/m^2^ and 30% of the women had body mass index < 18.5 kg/m^2^. We had data on urinary lead concentrations for 125 individuals and data on erythrocyte lead concentrations for 117 individuals. The maternal concentrations of lead varied 20-fold in urine (ranging from 0.81 to 17 µg/l, adjusted for specific gravity) and 10-fold in erythrocytes (ranging from 26 to 300 µg/kg erythrocytes) and both showed skew distributions. The concentrations of lead in erythrocytes and urine of the studied women did not differ from the concentrations among all women in the sub-cohort of 500 women that had measurements on lead concentrations (*P* = 0.32 and 0.78, respectively) [16]. The correlation between lead concentrations in erythrocytes and urine was *r* = 0.44 (*P* < 0.001). 

**Table 1:** characteristics of the 127^a^ mother–child pairs included in the study

Variable	Median (5–95th percentiles) or *N* (%)
Maternal characteristics	
Age (years)	25 (17–36)
BMI (kg/m^2^; GW8)	20 (17–29)
Betel chewing in pregnancy, yes/no (yes %)	73/52 (58/42%)
Urinary lead concentrations (µg/l; GW8)^b^	3.1 (1.4–8.6)
Quartile 1	1.7 (0.9–2.2)
Quartile 2	2.6 (2.4–3.1)
Quartile 3	3.7 (3.2–4.6)
Quartile 4	5.9 (4.6–15)
Erythrocyte lead concentrations (µg/kg; GW14)	94 (42–196)
Quartile 1	49 (29–68)
Quartile 2	83 (96–94)
Quartile 3	113 (96–131)
Quartile 4	168 (132–279)
Urinary arsenic metabolites (µg/l; GW8)^b^	68 (20–440)
Blood cadmium (µg/kg; GW14)	1.3 (0.6–3.0)
Newborn characteristics	
Boys/girls	62/65 (49/51%)
Gestational age at birth (weeks)	39 (36–42)
Birth weight (g)	2800 (220–3300)

BMI, body mass index; *N*, number of individuals; GW, gestational week.

^a^Number of individuals with data on urinary lead concentrations was 125 and with data on erythrocyte lead concentrations was 117.

^b^Adjusted to average specific gravity of 1.012.

### Differentially Methylated Positions Associated with Lead

**Figure 1:**
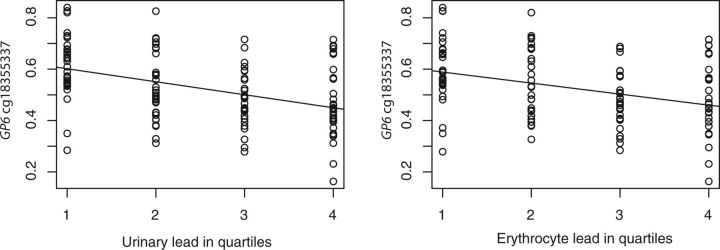
concentrations of lead in urine and in erythrocytes (both divided into quartiles) is inversely associated with cord blood methylation (showed as Beta-values) of the CpG site cg18355337 in *GP6*

Maternal lead in urine (divided into quartiles) was significantly [after adjustments for false discovery rate (FDR)] associated with the methylation status of nine CpGs, and for all these CpGs, increasing lead in urine was associated with decreasing methylation of DNA obtained from cord blood samples at delivery (Table 2). Three of these sites are located in the 5′-end of the glycoprotein IV gene (*GP6*) on chromosome 19: cg18355337 (Fig. 1) (*q* = 0.029, *β* = − 0.30), cg25818583 (*q* = 0.047, *β* = −0.17), and cg23796967 (*q* = 0.041, *β* = −0.18). In addition, lead in urine was near-significantly associated with an additional CpG site in *GP6* (cg05374025, *q* = 0.057, *β* = −0.23). These four CpG sites in *GP6* are located close to each other either in the promoter (cg23796967) or the promoter-flanking region. The CpGs in *GP6* were also associated with decreasing methylation with increasing lead in erythrocytes (divided into quartiles), although this had lower magnitude compared with lead in urine and was not statistically significant after adjustment for FDR: cg18355337 (unadjusted *P* = 8×10^−^^6^, *q* = 0.20, *β* = −0.25), cg25818583 (unadjusted *P* = 2×10^−^^4^, *q* = 0.40, *β* = −0.15), cg23796967 (unadjusted *P* = 6×10^−^^5^, *q* = 0.32, *β* = −0.14), and cg05374025 (unadjusted *P* = 2×10^−^^4^, *q* = 0.41, *β* = −0.18). 

**Table 2:** differentially methylated positions in cord blood in relation to quartiles of maternal urinary lead concentrations in early gestation

CpG	Chr.	Position^a^	Gene	Gene names	*β* ^b^	95% CI lower	95% CI higher	*q*	Beta^c^
cg18355337	19	55549722	*GP6*	glycoprotein VI (platelet)	–0.30	–0.42	–0.20	0.029	0.53
cg25196158	1	214152979	NA^d^ (*PROX1*)	(prospero homeobox 1)	–0.14	–0.20	–0.09	0.029	0.09
cg16943697	2	120280763	*SCTR*	secretin receptor	–0.10	–0.14	–0.06	0.029	0.54
cg03833077	19	10024709	*OLFM2*	olfactomedin 2	–0.32	–0.45	–0.20	0.029	0.19
cg12504721	5	138897583	NA (*TMEM173*)	(transmembrane protein 173)	–0.10	–0.15	–0.07	0.031	0.04
cg23796967	19	55549590	*GP6*	glycoprotein VI (platelet)	–0.18	–0.25	–0.10	0.041	0.72
cg00145875	2	11104394	NA (*KCNF1*)	(potassium channel, voltage gated modifier subfamily F, member 1)	–0.09	–0.12	–0.06	0.047	0.80
cg25818583	19	55549801	*GP6*	glycoprotein VI (platelet)	–0.17	–0.23	–0.10	0.047	0.63
cg23173307	4	786244	*CPLX1*	complexin 1	–0.14	–0.20	–0.09	0.047	0.04
cg04942107	10	3918567	NA (*KLF6*)	(kruppel-like factor 6)	0.07	0.04	0.08	0.057	0.87
cg05374025	19	55549746	*GP6*	glycoprotein VI (platelet)	–0.23	–0.34	–0.15	0.057	0.44
cg26668675	6	31148463	NA (*PSORS1C3*)	(psoriasis susceptibility 1 candidate 3)	–0.17	–0.23	–0.10	0.062	0.44
cg25472897	8	145560555	*SCRT1*	scratch family zinc finger 1	–0.10	–0.15	–0.06	0.070	0.18
cg00436174	2	128051630	*ERCC3*	excision repair cross-complementation group 3	−0.07	−0.12	−0.04	0.081	0.10
cg11790196	14	89995679	*FOXN3*	forkhead box N3	−0.29	−0.40	−0.17	0.089	0.95

Chr, chromosome; *q*, FDR-adjusted *P* value; CI, confidence interval.

^a^Position according to the Genome Reference Consortium GRCh37 [17].

^b^ *β* denotes *β*_1_ in the following robust regression model: *M* value = *β*_1_ × quartile lead in urine + *β*_2_ × surrogate variable (from sva analysis).

^c^The average methylation as Beta value for all individuals (ranging from 0 to 1, where 1 means fully methylated).

^d^NA, not applicable, i.e. the CpG is not present in any known gene, the gene name in brackets denotes the gene with the closest transcription start site.

Other CpGs that were significantly associated with urinary lead were situated in the secretin receptor (*SCTR*), olfactomedin 2 (*OLFM2*), complexin 1 (*CPLX1*), as well as three CpGs in intergenic regions for which the closest transcription start sites belonged to the genes prospero homeobox 1 (*PROX1,* 8306 basepairs 5′ of the CpG), transmembrane protein 173 (*TMEM173,* 35 240 basepairs 5′ of the CpG), and potassium channel, voltage-gated modifier subfamily F, member 1 (*KCNF1,* 52 332 basepairs 3′ of the CpG).

Maternal lead in erythrocytes was significantly (after FDR-adjustments) associated with the methylation status of two CpGs (Table 3): one CpG in fragile histidine triad (*FHIT*) on chromosome 3 (*q* = 0.001, *β* = −0.10) and one CpG in an intergenic region of chromosome 22, where the closest transcription start site (2898 basepairs 5′ of the CpG) belonged to the gene Shisa family member 8 (*SHISA8*) (*q* = 0.002, *β* = −0.11). 

**Table 3:** differentially methylated positions in cord blood in relation to quartiles of maternal erythrocyte lead concentrations in early gestation

CpG	Chr.	Position^a^	Gene	Gene names	*β* ^b^	95% CI lower	95% CI higher	*q*	Beta^c^
cg01519017	3	61227050	*FHIT*	fragile histidine triad	−0.10	–0.13	–0.07	0.001	0.90
cg16653408	22	42313569	NA^d^ (*SHISA8*)	(shisa family member 8)	–0.11	–0.15	–0.07	0.002	0.66
cg03978169	6	33091357	*HLA-DPB2*	HLA-DPB2	–0.12	–0.17	–0.08	0.052	0.90
cg18461584	7	55304314	NA (*EGFR*)	(epidermal growth factor receptor)	–0.13	–0.18	–0.08	0.089	0.92
cg23352003	18	24237245	NA (*KCTD1*)	(potassium channel tetramerization domain containing 1)	–0.29	–0.41	–0.18	0.13	0.03
cg07313720	2	209029594	*CRYGA*	crystallin, gamma A	0.29	0.18	0.41	0.14	0.95
cg04432660	7	4802132	*FOXK1*	forkhead box K1	–0.12	–0.17	–0.07	0.19	0.94
cg04606773	10	134759693	NA (*C4AP46*)	(cilia and flagella associated protein 46)	–0.15	–0.21	−0.09	0.19	0.91
cg22994018	11	105448797	NA (*GRIA4*)	(glutamate receptor, ionotropic, AMPA 4)	0.42	0.25	0.59	0.19	0.89
cg25979157	19	53902687	*ZNF765*	zinc finger protein 765	−0.09	−0.13	−0.05	0.19	0.92
cg11774346	21	46797899	NA (*COL18A1*)	(collagen, type XVIII, alpha 1)	−0.12	−0.17	−0.07	0.19	0.90
cg11668188	1	7891116	*PER3*	period circadian clock 3	0.45	0.26	0.64	0.19	0.96
cg25192902	11	121163419	*SC5DL*	sterol-C5-desaturase	−0.12	−0.17	−0.07	0.19	0.03
cg24737570	2	11727173	*GREB1*	growth regulation by estrogen in breast cancer 1	−0.07	−0.1	−0.04	0.19	0.82
cg05749559	8	36793457	*KCNU1*	potassium channel, subfamily U, member 1	−0.11	−0.16	−0.06	0.19	0.89

Chr, chromosome; *q*, FDR-adjusted *P* value; CI, confidence interval.

^a^Position according to the Genome Reference Consortium GRCh37 [17].

^b^ *β* denotes *β*_1_ in the following robust regression model: *M* value = *β*_1_ × quartile lead in erythrocytes + *β*_2_ × surrogate variable (from sva analysis).

^c^The average methylation as Beta value for all individuals (ranging from 0 to 1, where 1 means fully methylated).

^d^NA, not applicable, i.e. the CpG is not present in any known gene, the gene name in brackets denotes the gene with the closest transcription start site.

None of the top genes (Tables 2 and 3) were significantly associated with concentrations of arsenic in urine or cadmium in blood. We observed no differences between boys and girls in direction and magnitude of the effect of lead in urine or erythrocytes on *GP6* methylation. Further, we found no differences in lead-related *GP6* methylation between individuals who did or did not chew betel nuts (data not shown). We also conducted sensitivity analyses, where we adjusted for other environmental variables that could potentially influence DNA methylation (supplementation group, betel nut chewing, urinary arsenic, and urinary cadmium). These did not particularly change the results, and the top hits for GP6 were still statistically significant.

### Differentially Methylated Regions Associated with Lead

**Figure 2:**
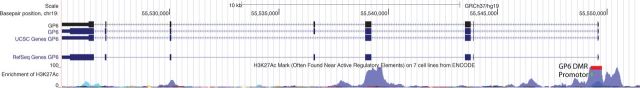
the *GP6* gene (derived from the UCSC genome browser) with illustrations of the promoter [19] (in green) and the DMR (in red) in relation to prenatal exposure to lead. The gene is transcribed in 3′–5′ direction (from the right to the left)

For lead in urine, 1010 candidate differentially methylated regions (DMRs) were found (Table 4), of which 861 were inversely associated with lead in urine (85%). For lead in erythrocytes, 626 candidate DMRs were found (Table 5), of which 468 were inversely associated with lead in urine (74%). However, after performing FDR adjustments, none of the DMRs was statistically significantly associated with lead in urine or erythrocytes. For urinary lead, the top hits were four regions in chromosome 6 and one region in chromosome 19, the latter including eight CpG sites in *GP6* (*P* = 0.005, *q* = 0.48), including all *GP6* top hits in the differentially methylated position analysis (Fig. 2). Some of the DMRs situated on chromosome 6 were in major histocompatibility complex genes, such as ring finger protein 39 (*RNF39*), psoriasis susceptibility 1 candidate 3 (*PSORS1C3*), and HLA complex group 4b (*HCG4P6*). Also for lead in erythrocytes, there was also one DMR of eight CpGs in *GP6* (*P* = 0.007, *q* = 0.46), and several of the top hits were in major histocompatibility complex genes on chromosome 6, including major histocompatibility complex class I H, class II DQ beta I, and DP beta II (*HLA-H, HLA-DQB1, HLA-DPB1*), HLA complex group 4B (*HCG4P6*), and lymphocyte antigen 6 complex, locus G5C (*LY6G5C*). 

**Table 4:** DMRs in cord blood in relation to quartiles of maternal urinary lead concentrations in early gestation

Gene	Gene names	Chr.	Start^a^	End^a^	Direction^b^	Area^c^	Nr. CpGs	*P* unadj.	*q*
*RNF39*	ring finger protein 39	6	30038998	30039600	+	4.05	26	0.0004	0.40
NA^d^ (*PSORS1C3*)	(psoriasis susceptibility 1 candidate 3)	6	31148332	31148748	–	1.98	15	0.003	0.48
*NFYA*	nuclear transcription factor Y, alpha	6	41068553	41068752	–	1.61	7	0.004	0.48
*CRISP2*	cysteine-rich secretory protein 2	6	49681178	49681391	–	1.43	7	0.005	0.48
*GP6*	glycoprotein VI (platelet)	19	55549414	55549842	–	1.42	8	0.005	0.48
*PIWIL2*	piwi-like RNA-mediated gene silencing 2	8	22132678	22133076	–	1.37	9	0.005	0.48
*C5orf63*	chromosome 5 open reading frame 63	5	126408756	126409372	–	1.34	11	0.005	0.48
*TYW3*	tRNA-yW synthesizing protein 3 homolog (*S. cerevisiae*)	1	75198211	75199117	–	1.29	11	0.006	0.48
NA (*ZBED9*)	zinc finger, BED-type containing 9	6	(28601269	28601519)	–	1.22	13	0.006	0.48
*KRTCAP3*	keratinocyte associated protein 3	2	27665017	27665711	–	1.15	11	0.007	0.48
*PSMA8*	proteasome (prosome, macropain) subunit, alpha type, 8	18	23713407	23714084	–	1.10	10	0.008	0.48
*SLFN12*	schlafen family member 12	17	33759512	33760293	–	1.06	10	0.009	0.48
*PAX8*	paired box 8	2	113992762	113992930	+	1.05	4	0.009	0.48
*PXDNL*	peroxidasin-like	8	52321814	52322171	–	1.04	6	0.009	0.48
*HCG4P6*	HLA complex group 4B	6	29894619	29894820	–	1.03	6	0.009	0.48

Chr, chromosome; *q*, FDR-adjusted *P* value.

^a^Position of start and end of the DMR according to Genome build 37 [17].

^b^Direction of association with urinary lead (as a continuous variable, divided into quartiles).

^c^The strength of evidence for each DMR is summarized with its area [18].

^d^NA, not applicable, i.e. the CpG is not present in any known gene, the gene name in brackets denotes the gene with the closest transcription start site.

**Table 5:** DMRs in cord blood in relation to quartiles of maternal erythrocyte lead concentrations in early gestation

Gene	Gene names	Chr	Start^a^	End^a^	Direction^b^	Area^c^	Nr. CpGs	*P* unadj.	*q*
NA^d^ (*VTRNA2-1*)	(vault RNA 2-1)	5	135415693	135416613	–	2.87	16	0.001	0.46
*GSTT1*	glutathione-S transferase T1	22	24384105	24384573	+	1.60	11	0.004	0.46
NA (*LY6G5C*)	(lymphocyte antigen 6 complex, locus G5C)	6	31650735	31651094	–	1.51	14	0.004	0.46
*HCG4P6*	HLA complex group 4B	6	29894619	29894946	–	1.50	8	0.005	0.46
*NFYA*	nuclear transcription factor Y, alpha	6	41068553	41068752	–	1.49	7	0.005	0.46
*LSMEM1*	leucine-rich single-pass membrane protein 1	7	112121036	112121259	+	1.47	2	0.005	0.46
*HLA-H*	major histocompatibility complex, class I, H	6	29855110	29856070	–	1.29	9	0.006	0.46
NA (*ZFP57*)	(ZFP57 zinc finger protein)	6	29648525	29649084	+	1.25	10	0.006	0.46
*GP6*	glycoprotein VI (platelet)	19	55549414	55549842	–	1.22	8	0.007	0.46
*AURKC*	aurora kinase C	19	57741988	57742444	–	1.11	10	0.008	0.46
*VWA7*	von Willebrand factor A domain containing 7	6	31744545	31745181	–	1.10	12	0.008	0.46
*HLA-DQB1*	major histocompatibility complex, class II, DQ beta 1	6	32632937	32633163	–	1.08	8	0.009	0.46
*HLA-DPB2*	major histocompatibility complex, class II, DP beta 2	6	33091242	33091841	–	0.96	8	0.011	0.53
*DPPA5*	developmental pluripotency associated 5	6	74063982	74064594	–	0.91	7	0.012	0.56
NA (*RPTN*)	(repetin)	1	152161237	152162025	–	0.86	7	0.014	0.57

Chr, chromosome; *q*, FDR-adjusted *P* value.

^a^Position of start and end of the DMR according to Genome build 37 [17].

^b^Direction of association with erythrocyte lead (as a continuous variable, divided into quartiles).

^c^The strength of evidence for each DMR is summarized with its area [18].

^d^NA, not applicable, i.e. the CpG is not present in any known gene, the gene name in brackets denotes the gene with the closest transcription start site.

### Validation of GP6 Methylation in Relation to Lead

The sites in *GP6* were validated by methylation-sensitive high-resolution melting analyses (MS-HRM) in a subset of the samples (*n* = 80, based on DNA availability) that had been analyzed by Illumina 450 K. The MS-HRM assay covered three sites in *GP6*: cg02462353, cg25818583, and cg20651389, all of which were present in the eight-CpG-long DMR of *GP6*. The MS-HRM analysis showed a significant, inverse correlation between maternal lead in urine or erythrocytes and the degree of DNA methylation (Spearman correlation, *r*_S_ = −0.37, *P* = 0.0008 for lead in urine and *r*_S_ = −0.22, *P* = 0.055 for lead in erythrocytes).

## Discussion

In this study including rural Bangladeshi women with moderate lead exposure, increasing lead in urine in early gestation was associated with decreasing DNA methylation for a number of CpG sites in cord blood of the newborns. In particular, the gene *GP6*, which codes for glycoprotein VI (protein name GPVI), was identified as a target for lead exposure, which is a novel finding. Urinary lead was significantly associated with three CpGs in the 5′ region of GP6 and eight CpG sites in *GP6* constituted a DMR in relation to lead in urine. One of these CpGs was present within the promoter (cg23796967) [19], and the others were situated in proximity to the promoter. This region also shows signs of active regulation of gene expression as judged by active histone marks (Fig. 2), indicating that lead-related DNA methylation in this region may affect the expression of *GP6*.

GPVI is a transmembrane platelet collagen receptor which functions as a dimer and is an important component of platelet activation [20]. Dimerization and activation of GPVI occur in response to damage in a blood vessel, resulting in recruitment of collagen and thrombocytes, which give rise to a platelet plug and, at later stages, coagulation by fibrin [21, 22]. Moreover, GPVI has been linked to cardiovascular diseases. Several studies have reported elevated levels of GPVI protein in whole blood of patients with acute coronary syndrome, acute ischemic stroke, transient ischemic attack, or stroke, compared with patients without ischemic conditions [23–26]. Platelets have no nucleus and derive from megakaryocytes in the bone marrow. We cannot tell whether the lead-related methylation of *GP6* found in cord blood in this study also reflects the methylation status of *GP6* in megakaryocytes in the bone marrow. However, *GP6* expression in cord blood as well as during megakaryocyte differentiation has been shown to be dependent on demethylation of the *GP6* promoter, where the demethylation correlated with increased mRNA levels [27]. Several studies have linked fatal and non-fatal cardiovascular events to exposure to lead; e.g. studies based on the NHANES II and III data showed strong associations between lead exposure and cardiovascular disease, coronary heart disease and stroke, and blood pressure [28–30]. Navas-Acien et al. [31] concluded that the available data indicate a causal relationship between lead exposure and elevated blood pressure in adults. On the basis of the present results, we hypothesize that the lead-related lower methylation of *GP6* in cord blood, and potentially in the bone marrow, very early in fetal development may result in increased levels of GPVI. When cardiovascular insult occurs later in life, for whatever reason, higher levels of GPVI might result in stronger platelet activation and increase the risk of cardiovascular problems. Thus, the epigenetic effect of lead early in life could substantially contribute to the overall adverse cardiovascular effects of lead poisoning later in life and could contribute to lead-induced hypertensive effects [32], antifibrinolytic effects [33], and the procoagulant activation of erythrocytes [34]. Interestingly, a recent study indicated that maternal postpartum bone lead concentrations were associated with increased blood pressure in their children at 10 years of age [35].

We also identified associations between lead in urine or erythrocytes and CpGs in other genes, in particular in the HLA region on chromosome 6, responsible for regulation of the immune system. However, little is known about the immunotoxicity of lead, especially in the general population. Alterations of immunoglobulin levels (e.g. IgE, IgG, and IgA) in serum or saliva, as well as various effects on leukocyte and lymphocyte subtypes have been seen in lead-exposed workers, although several studies found no associations [36]. Furthermore, an earlier study on Japanese lead refinery workers indicated that lead exposure is associated with increased sensitivity to infections [37]. The potential effect of lead on the immune system is an interesting finding, and the immune response among lead-exposed children should be further characterized. Further, the CpG that showed the strongest association with lead in erythrocytes was situated in the fragile histidine triad gene (*FHIT*), a tumor suppressor gene located in a common fragile site that can be rearranged due to exposure to carcinogens. *FHIT* plays an important role in lung cancer development [38]. However, its role in lead-related carcinogenesis is unknown.

There were a larger number of statistically significant findings for lead measured in urine, measured very early in pregnancy (gestational week, GW, 8), compared with lead measured in erythrocytes, measured somewhat later in pregnancy (GW14). It is not clear if the timing of lead exposure influenced the effect of lead on DNA methylation. Lead in urine is considered a short-term marker, whereas lead in erythrocytes reflects approximately the last month of exposure [39]. Also, the erythrocyte lead concentrations might have been influenced by early plasma expansion in pregnancy. Another possibility is that the study included somewhat fewer individuals with data on lead in erythrocytes (*N* = 117), compared to those with data on lead in urine (*N* = 125), resulting in lower power for associations with erythrocyte lead.

The median concentration of lead in maternal erythrocytes was 94 μg/kg, corresponding to 4 μg/dl in whole blood, assuming 99% of blood lead in the erythrocytes, 41% hematocrit, and an erythrocyte density factor of 1.06 kg/l. These levels are similar to those observed in pregnant women in China and Mexico [40, 41] and higher than those measured in pregnant women in Canada and Germany [42, 43].

One possible drawback of this study is that we were not able to separate cells from blood samples in the field. Therefore, we measured DNA methylation in mixed mononuclear cells from cord blood, i.e. cells that may have partly different methylation patterns. Thus, there is a possibility that a potential cell-specific effect of lead might blur associations between DNA methylation and prenatal lead exposure. However, we performed surrogate variable analysis to adjust for sources of noise such as variation in DNA methylation from different cell types. Importantly, the general lead-associated decreased methylation of specific genes identified here is in accordance with previous studies in newborns [44] and adults [45, 46]. We also conducted sensitivity analyses, in which we adjusted for environmental factors. However, other environmental factors for which we have no data, such as prenatal stress, may influence methylation status in newborns [47].

In conclusion, lead exposure early in life was associated with decreased DNA methylation of *GP6,* a key component in platelet activation. Decreased methylation of this gene might be a mechanism for lead-related cardiovascular disease. The functional significance of alterations in gene expression and protein levels due to changes in methylation in *GP6* is not known, and further studies are needed to clarify this.

## Methods

### Study Area and Design

This study was conducted in a non-industrialized, rural area called Matlab, located about 50 km southeast of Dhaka, Bangladesh, where moderate lead exposure (range of blood lead concentrations, calculated from erythrocyte lead concentrations, in early pregnancy was 1.4–11.5 µg/dl) has been documented [16]. Main lead sources were suggested to be food and drinking water, cooking pots, and tin roofs and walls, commonly used in the area. In Matlab, the International Centre for Diarrheal Disease Research, Bangladesh (icddr,b) operates a central hospital and four connected health care facilities, as well as a health and demographic surveillance system, which is updated monthly with information collected by community health research workers.

This study was nested in a large randomized food and micronutrient supplementation trial conducted during pregnancy [Maternal and Infant Nutrition Interventions in Matlab (MINIMat)] [48]. In total, 4436 pregnant women were found to be eligible (viable fetus, gestational age < 14 weeks, no severe illness, and consent for participation) and thereafter enrolled in the MINIMat trial from November 2001 to October 2002. The supplementation in the intervention trial consisted of food supplementation (starting in early- or mid-pregnancy) and one of three different micronutrient supplementations (provided daily from GW 14): (i) 30 mg iron and 400 mg folic acid; (ii) 60 mg iron and 400 mg folic acid; or (iii) a capsule with 15 micronutrients, including 30 mg iron and 400 mg folic acid [49].

For this study, we selected a subset of 127 women who were enrolled in the MINIMat trial from October 2002 to October 2003, gave birth to a singleton child at the health care facilities during early day time, and had cord blood collected at delivery [50]. The main reasons for the low number of women in the present subset are a high frequency of home deliveries (>60 %) and that many deliveries at the health care facilities occurred in late afternoon or night, when the logistics did not allow for processing and transport of samples to the laboratory in Dhaka. When comparing these 127 women with all other women who were enrolled in the MINIMat trial from October 2002 to October 2003 and gave birth to a single child (*n* = 1729), the subset of 127 women had slightly higher socioeconomic status and were more likely to be primiparous [51]. Of the 127 mother–child pairs, 125 had data on maternal lead concentrations in urine, and 117 had data on maternal lead concentrations in erythrocytes. The study was approved by the ethics committees at icddr,b (Dhaka, Bangladesh) and the Karolinska Institutet (Stockholm, Sweden). Consent was obtained from all participants, and participants were free to refrain from any part of the study at any time.

### Analysis of Lead

Lead exposure during pregnancy was assessed based on concentrations in maternal urine collected at the time of detection of pregnancy by urine sticks, i.e. at about GW8, and in maternal erythrocytes collected at supplementation baseline, about GW14. Lead concentrations in urine and erythrocytes were measured by inductively coupled plasma mass spectrometry (Agilent 7500ce, Agilent Technologies, Japan), with the collision/reaction cell system operating in standard mode (no gas), at the Karolinska Institutet, Stockholm. The sample preparation method and a detailed description of the inductively coupled plasma mass spectrometry analyses have been previously published [16, 52, 53]. All samples had detectable levels of lead. Quality control was assessed by analyses of two commercial control materials (Seronorm Trace Elements Urine Blank, REF 201305, LOT OK4636 and Seronorm Trace Elements Urine, REF 201205, LOT NO2525). To compensate for variations in the dilution of urine, concentrations were adjusted to the mean specific gravity for mothers’ urine (1.012) measured using a digital refractometer (EUROMEXRD712 clinical refractometer; EROMEX, Arnhem, Netherlands) [54]. Prenatal exposure to arsenic and cadmium (maternal sum of arsenic metabolite concentrations in urine and maternal cadmium concentrations in blood) were measured in GW8 as described previously [51, 55].

### Analysis of DNA Methylation

Umbilical cord blood (mixed arterial and venous blood) was collected at birth in heparin-coated sterile vials (Becton Dickinson, Stockholm, Sweden) at the health care facilities. Mononuclear cells were separated by Ficoll (Pharmacia-Upjohn/McNeill Laboratories, NJ) density gradient centrifugation. DNA was isolated using QIAamp DNA Blood Midi kit (Qiagen, catalog number 51185). The DNA quality was evaluated with a NanoDrop spectrophotometer (NanoDrop Products, Wilmington, DE) and Bioanalyzer 2100 (Agilent, Santa Clara, CA) and showed good quality (260/280 nm >1.80). One microgram of DNA was bisulfite treated with the EZ DNA Methylation kit (Zymo Research, catalog number D5002). The DNA samples were randomized for distribution in the analysis plates (96-well plates); thus, there were no differences in gender distributions or lead in urine and erythrocytes between the plates. For each sample, 200 ng of DNA was used for methylation assessment with the Infinium HumanMethylation 450 K BeadChip (lllumina, catalog number WG-314-1002). All beadchips were from the same batch. Image processing, background correction, quality control, filtering, and normalization (by the SWAN procedure) were performed in the R package minfi. The 450 k included a total of 485 512 sites before filtering. All samples performed well, since they all had at least > 98% of the CpGs with detection *P* value below 0.01. We removed CpGs for which more than 20% of the samples had a detection *P* value above 0.01 (*N* = 1117). Furthermore, the following probes were removed: rs probes [probes that measure single-nucleotide polymorphisms (SNPs) but not probes containing SNPs] and CpH probes (representing nonCpG methylation) (*N* = 3091), probes with *in silico* nonspecific binding (*N* = 29 118) [56], probes on the X and Y chromosomes (*N* = 10 329), and probes with common SNPs (according to the function dropLociWithSnps in minfi; *N* = 15 424). In total, 426 433 probes were left for further analysis.

To validate associations between lead and specific CpG sites, a subset (*n* = 80; Supplementary Material and Table S1) of cord blood DNA samples with enough of DNA for validation was analyzed by MS-HRM analysis [57]. This subset did not differ in characteristics from the larger study group (Supplementary Table S1). Primers were designed for target CpGs with MethPrimer (urogene.org/methprimer/): (forward) 5′-ACGGGAATATAGATTAGGTTTTAGTAG-3′ and (reverse) 5′CGTAACCGACTCCTCAATACAATA-3′ (primers from TAG Copenhagen A/S, Denmark). The primer hybridization temperature was estimated by OligoCalc (basic.northwestern.edu/biotools/oligocalc.html). Fully methylated and unmethylated control DNA samples were obtained from Zymo (Zymo Research, catalog number D5014) and Qiagen (EpiTect Control DNA, catalog number 59695), respectively. Bisulfite-treated cord blood DNA (3 µl) was mixed with primers (0.5 µl of each primer, 500 nM final concentration), LightCycler 480 High Resolution Melting Master Mix (5 µl) (Hoffmann-La Roche, catalog number 04909631001) and MgCl_2_ (1.2 µl, 2.5 mM) and the polymerase chain reaction analysis was performed with a LightCycler 480 (Hoffmann-La Roche, Basel, Switzerland). A standard curve was included with control DNA methylated to different extents (100, 10, 1, 0.1, and 0%). The polymerase chain reaction amplifications were run at 52°C for 50 cycles and then at 90°C to denature the products.

### Statistical Analysis

After filtering, 426 433 probes were left for further analyses. Principal component analysis (PCA) was performed to evaluate the influence of technical and biological variables on DNA methylation and thus should be considered as covariates in the models. For the PCA, we employed the universally applicable singular value decomposition. The PCA was first performed on DNA methylation values expressed as raw normalized *M* values, and it showed that the *M* values were associated with analysis plate in the first and second principal components (PCs) (Supplementary Fig. S1, upper panel). These PCs explained 20% of the variance. The data were then adjusted for analysis plate via ComBat adjustment [58]. Second, we performed a new PCA on the ComBat-adjusted *M* values, where most of the variation due to methylation plate was removed (Supplementary Fig. S1, lower panel). There were no other variables identified with a major impact on DNA methylation based on inspections of the plots showing associations with the PCs (Supplementary Fig. S1, lower panel). ComBat-adjusted *M* values were then used for downstream analyses.

Cord blood contains different types of cells, and cell composition could impact the DNA methylation. The method by Houseman et al. [59], which often is used for estimating cell composition, is based on adult white blood cells as a reference. We instead performed surrogate variable analysis using the “sva” package to remove unwanted variation, such as variation in cell composition, by making surrogate variables that were used in subsequent analyses to adjust for unknown, unmodeled, or latent sources of noise [60]. The sva is closely related to the reference-free EWAS method by Houseman et al. [61].

In the downstream analyses, individuals were grouped into quartiles according to their concentrations of lead in urine or lead in erythrocytes, due to skewed distributions of raw data (also when lead concentrations were natural log transformed) as evident from inspection of histograms. The quartiles for urinary lead and erythrocyte lead, respectively, were used as continuous variables (labelled 1–4). Differentially methylated positions were evaluated by fitting a robust linear regression model to each CpG using the R package limma with adjustment for the surrogate variable obtained from sva. Empirical Bayes smoothing was applied to the standard errors. *P* values were adjusted for multiple comparisons by the Benjamini–Hochberg FDR method [62] to obtain *q*-values. A *q*-value of 0.05 or lower was considered statistically significant. Because we have previously shown that this study population is exposed to arsenic and cadmium [63, 64] and arsenic and cadmium exposure has been associated with DNA methylation [51, 55], we also evaluated whether the top genes were associated with maternal arsenic concentrations in urine (natural log transformed, measured in GW 8) or cadmium concentration in blood (natural log transformed, measured in GW14) (adjusting for surrogate variables obtained). Urine and blood samples were collected prior to food and micronutrient supplementation; the cord blood samples for analysis of DNA methylation were collected at delivery (thus after the supplementation started). We have data on supplementation in a variable called “supplementation group,” including six different groups, which combines data on supplementation of food [two groups: (i) early invitation at GW 9 or (ii) usual invitation at ∼GW20] and micronutrient supplements (two groups with different combinations of iron and folic acid, and one group with iron, folic acid, and 13 additional micronutrients) [48]. We evaluated the data stratified for sex or betel nut chewing (yes/no). Finally, we also conducted sensitivity analyses, where we adjusted for other environmental variables that could potentially influence DNA methylation. Variables included in the analyses were arsenic concentrations in urine (natural log transformed, measured in GW 8), cadmium concentration in blood (natural log transformed, measured in GW14), betel nut chewing (yes/no), as well as supplementation group.

DMRs were evaluated using bumphunter, which also estimated potential unmeasured confounders via sva [18], in the R package minfi. The maximum gap between the CpGs was set to 300 basepairs. The smoothing function was employed and 1000 iterations were performed.

All statistical analyses were performed using R (v.3.1.3).

## Supplementary Material

Supplementary Data Supplementary Data
