# Supplementary material for: Prenatal lead exposure is associated with decreased cord blood DNA methylation of the glycoprotein VI gene involved in platelet activation and thrombus formation
Source: Environ Epigenet. 2015 Nov 27;1(1):dvv007. doi: 10.1093/eep/dvv007 (PMC5804686; doi:10.1093/eep/dvv007)
Supplement: Supplementary Data [file dvv007_supplementary_data.zip › Supplemental_Engstrometal.docx]

**Supplemental Material for:**

**Prenatal lead exposure is associated with decreased cord blood DNA methylation of the glycoprotein VI gene involved in platelet activation and thrombus formation**

Karin Engström, Filip Rydbeck, Maria Kippler, Tomasz K Wojdacz, Shams Arifeen, Marie Vahter, Karin Broberg

**Table of contents:**

Supplemental Material, Table S1. Characteristics of the sub-sample of newborns used for validation of DNA methylation results from the 450K beadchips.

Supplemental material, Figure S1. Overview of associations (by a linear model) of the principal components with technical and biological variables*.* The upper panel shows data from the first principal component analysis (PCA) containing raw normalized M-values, while the lower panel contains data from the second PCA containing normalized M-values that are also adjusted for analysis plate (ComBat).

Supplemental Material, Table S1. Characteristics of the sub-sample of 80 newborns used for validation of DNA methylation results from the 450K beadchips.

| **Variable** | **Sub-sample (N=80)^a^** |
| --- | --- |
| *Maternal characteristics* |  |
| Age (years) | 25 (16-37) |
| BMI (kg/m^2^; GW8) | 20 (17-26) |
| Betel chewing in pregnancy (yes/no) (yes%) | 44 (56 %)/34 (44 %) |
| Urinary lead concentrations (µg/L; GW8)^b^ | 3.1 (1.3 - 8.8) |
| Erythrocyte lead concentrations (µg/kg; GW14)^b^ | 90 (36 – 186) |
| Urinary arsenic (µg/L; GW8)^b^ | 107 (25-526) |
| Blood cadmium (µg/kg; GW14)^c^ | 1.34 (0.56 - 3.0) |
| *Newborn characteristics* | |
| Boys/girls | 40/40 (50/50 %) |
| Gestational age at birth (weeks) | 39 (36-41) |
| Birth weight (g) | 2735 (2140- 3270) |

Abbreviations: BMI, Body mass index; GW, Gestational week; N, Number of individuals.

^a^Values are shown median (5-95 percentiles) or n (%).

^b^Adjusted to average specific gravity of 1.012.

^c^Available for n=75.


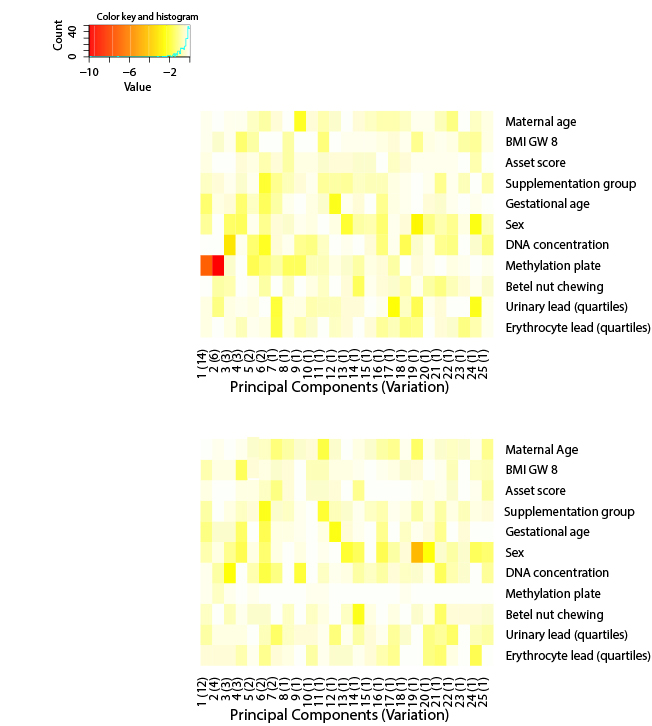


Supplemental material, Figure S1. Overview of associations (by a linear model) of the principal components with technical and biological variables*.* The upper panel shows data from the first principal component analysis (PCA) containing raw normalized M-values, while the lower panel contains data from the second PCA containing normalized M-values that are also adjusted for analysis plate (ComBat). Abbreviations: BMI, Body mass index; GW, Gestational week.
